# Reverse LISS plating for intertrochanteric Hip Fractures in elderly patients

**DOI:** 10.1186/1471-2474-11-166

**Published:** 2010-07-21

**Authors:** CQ Zhang, Y Sun, DX Jin, C Yao, SB Chen, BF Zeng

**Affiliations:** 1 Department of Orthopedics, Shanghai Sixth People's Hospital, Shanghai Jiao Tong University, 600 Yishan Road, Shanghai 200233, PR China

## Abstract

**Background:**

Fractures of the intertrochanteric hip are common and the treatment of unstable fractures generally requires an operative approach. In elderly patients, osteoporosis makes internal fixation problematic and frequently contributes to failed fixation and poor clinical results. We have attempted to apply the Less Invasive Stabilization System (LISS) in reverse position for the repair of intertrochanteric hip fractures in elderly patients with osteoporotic bones. A retrospective review is presented of the cases of 28 elderly patients with stable and unstable fractures of the intertrochanteric hip treated using the reverse LISS.

**Methods:**

We treated 28 elderly patients with a mean age of 82.3 years. According to the Evens classification, there were 2 Type I fractures, 2 Type II fractures, 3 Type III fractures, 13 Type IV fractures, 6 Type V fractures and 2 Type R fractures. All fractures were treated using the reverse LISS. Radiographic and clinical evidence of functional outcome and complications were evaluated.

**Results:**

Mean perioperative blood loss was 92.4 milliliters (range 35 to 245 milliliters), and the mean postoperative hospital stay was 8.7 days (range 3 to 14 days).

Complications included one minor wound hematoma. Radiographically, no collapses, screw cutouts, or head penetrations were seen. All surviving patients (28 of 28; 100 percent) had uneventful fracture healing with union achieved by six months in all patients.

**Conclusions:**

Use of the Reverse LISS plating for intertrochanteric hip fractures resulted in event-free fracture healing.

## Background

Hip fractures are a leading cause of death and disability among the elderly. Approximately 50% of hip fractures are intertrochanteric fractures, a large percentage of which are unstable [1,2]. Treatment goals for this patient population include early rehabilitation, restoration of the anatomic alignment of the proximal part of the femur, and maintenance of the fracture reduction [3]. Different approaches have been used to solve this problem, including trochanteric osteotomy techniques, cementing, and different types of fixation devices. Despite improved techniques and devices, failure of fixation is still a problem in unstable intertrochanteric fractures[4].

In recent years, the minimally invasive surgical techniques have led to a widespread use of many new implants [5,6]it has been shown that they can reduce operative complications and postoperative morbidity. As such, the present study evaluates the treatment of intertrochanteric hip fractures with the reverse LISS plating system.

## Methods

The present study was reviewed and approved by our institutional review board, and informed consent was obtained from all patients. The patients provided were informed for use of their clinical images. Twenty-eight patients with intertrochanteric fractures underwent the reverse LISS procedure and were reviewed retrospectively. Fractures were classified according to the Evens classification [7]. Routine investigation on admission to the hospital included assessment of coexisting medical conditions, blood electrolyte and urea monitoring, complete blood cell count, electrocardiography, and chest radiography. Attempts were made to stabilize preexisting conditions before surgery. Closed fracture reduction was performed using the fracture table under image intensifier control. Traction and rotation were used to achieve and maintain reduction during the surgical procedure as seen in the anteroposterior and lateral views.

## Surgical Technique

**Figure 1:**
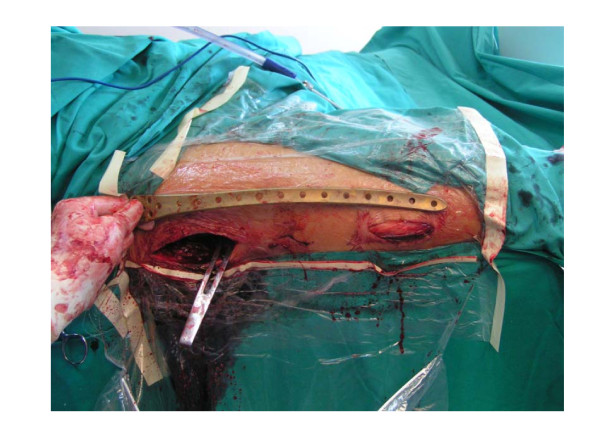
**Implant introduced through separate proximal and distal incisions**.

After basic fracture reduction, a short proximal incision was made over the greater trochanter (Figure 1) and an appropriate-hole LISS plate was chosen (As usual, for Type I, II, III fractures, a 9-hole LISS plate was chosen; for reverse oblique and transverse Intertrochanteric fractures with/without femoral fractures, a longer LISS plate should be chosen, for example a 13-hole LISS plate). An implant of the contralateral limb was chosen in order to accommodate the anterior bow of the femur (i.e. a left sided LISS plate was to be used "upside down" for the right femur). The plate was then introduced through the proximal incision and was slid down distally beneath the muscle tissue without stripping the periosteum of the lateral femur. Subsequently, the plate was maneuvered onto the distal fragment through a short distal incision, using bone-holding forceps. In this position, proper placement of the plate, frontal and rotational alignment and leg length were checked. If there was any malalignment, rotational deformity or limb-length discrepancy, reduction was repeated after releasing the bone holding forceps in the distal fragment. After reduction and proper placement of the plate and before distal fixation, proximal locking screws were then passed through the threaded screw hole of the normally distal part of the plate and up the centre of the neck. Satisfactory position was then checked on AP and the lateral planes. Following this, distal fixation was performed through the distal incision and the operation was checked radiographically. Generally, four locking screws were placed in the proximal fragment; four in the distal. Stabilization was achieved within the 35 min time frame. The wound is irrigated and closed over a suction drain.

On the second postoperative day, range of movement exercises and straight leg lifts were started. On the third postoperative day, patients began walking using crutches or a frame, with touch-down weight-bearing. Progressive weight-bearing was encouraged. One month after surgery, the patients were mobilized and full weight-bearing weight bearing without any limitations.

All patients received perioperative prophylactic antibiotics until after removal of the drains. Intraoperative and postoperative blood loss, complications, postoperative ambulation, and length of stay in the hospital were recorded. Postoperative radiographs were assessed for fracture reduction and position of the plate and screws. Patients were examined clinically and radiographically at three, six, and twelve months, with a minimum follow-up period of twelve months.

## Results

**Figure 2:**
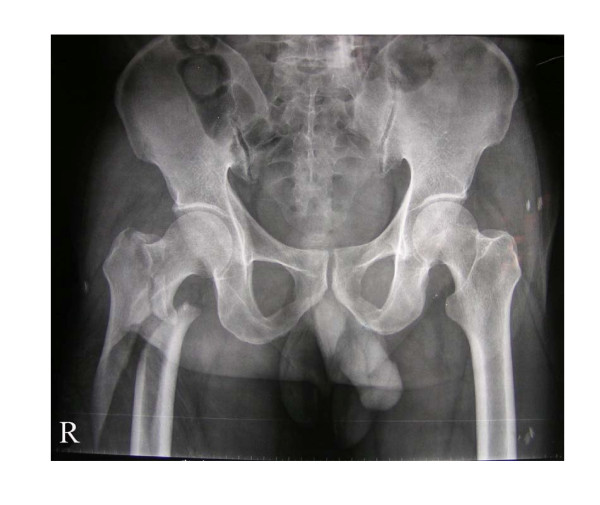
**Initial radiograph of one representative case showing unstable intertrochanteric and subtrochanteric fractures of the right femur**.

**Figure 3:**
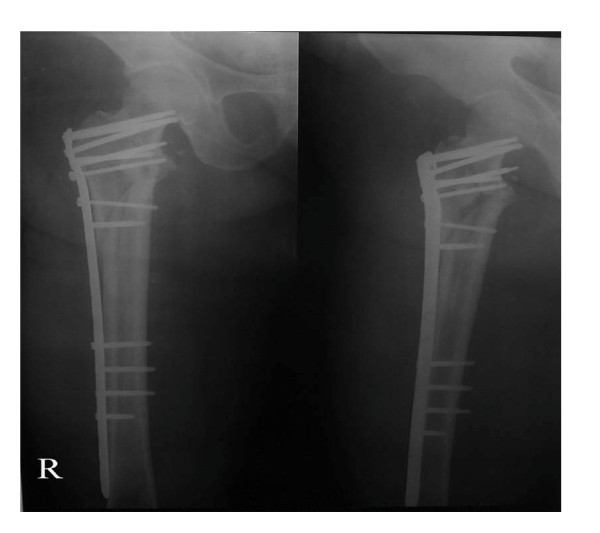
**Postoperative AP and lateral Radiographs of the unstable intertrochanteric fracture stabilized by reverse LISS plating**.

**Figure 4:**
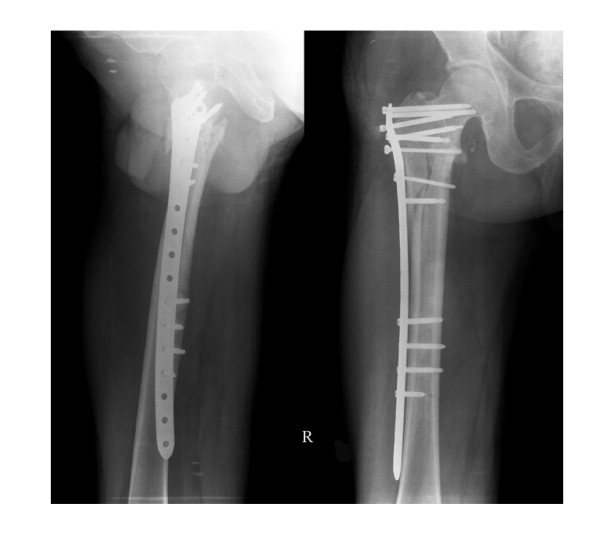
**Lateral radiograph and AP radiograph at 4 weeks post fixation showing callus formation**.

**Figure 5:**
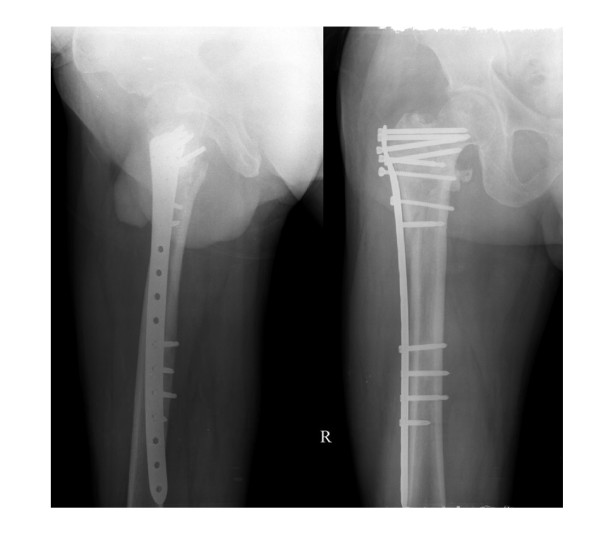
**AP and lateral radiograph at 16 weeks post fixation showing union of the fractures**.

According to the Evens classification[7], there were 2 Type I fractures, 2 Type II fractures, 3 Type III fractures, 13 Type IV fractures, 6 Type V fractures and 2 Type R fractures. Mean patient age was 82.3 years (range 58 to 102 years), and patients included 19 women and 9 men. No patients were lost to follow-up or died during the period of follow-up. Mean perioperative blood loss was 92.4 milliliters (range 35 to 245 milliliters), and the mean postoperative hospital stay was 8.7 days (range 3 to 14 days). Two patients had bronchopneumonia; one patient had a minor wound hematoma. Deep vein thrombosis, pulmonary embolus or operative wound complications were not observed. On radiological follow-up, there was one mild varus deformity of 8 degrees. There were no collapses, cutouts, or screw penetrations, but backing out and loosing of the locking screws were observed in 2 cases. In all patients, uneventful fracture healing and union was achieved by six months (Figures 2, 3, 4 and 5). No patient had poor functional result or failure, and all were satisfied with their postoperative functional results at the latest follow-up (Table 1).

**Table 1:** Patient demographics

Case	Gender	Age(years)	Type of fracture (Evens)	implant	Follow-up(months)
1	F	77	I	LISS	14
2	F	83	V	LISS	22
3	F	58	IV	LISS	27
4	M	88	IV	LISS	17
5	F	85	V	LISS	34
6	F	84	IV	LISS	32
7	F	82	IV	LISS	28
8	M	87	II	LISS	19
9	M	82	IV	LISS	29
10	F	83	IV	LISS	33
11	F	81	V	LISS	23
12	F	75	III	LISS	18
13	M	87	IV	LISS	21
14	M	79	R	LISS	13
15	F	83	IV	LISS	16
16	F	102	IV	LISS	12
17	F	91	IV	LISS	18
18	F	76	I	LISS	27
19	F	80	III	LISS	16
20	M	79	IV	LISS	21
21	M	84	IV	LISS	20
22	F	86	V	LISS	15
23	F	84	V	LISS	13
24	F	83	IV	LISS	18
25	M	78	V	LISS	15
26	F	71	R	LISS	21
27	F	88	III	LISS	15
28	M	89	II	LISS	14

M/F, male/female;

## Discussion

The "LISS" system was developed by the AO group for stabilization of distal femur and proximal tibial fractures. It is an extramedullary internal fixator which combines the advantages of both interlocked intramedullary nailing techniques and the early advances of the so-called biological plating technique into one system [8]. Although there is scarce literature regarding the use of reverse LISS plates for the treatment of unstable intertrochanteric fractures, the application of LISS have shown early promising results in periprosthetic fracture of the proximal femur. In a recent study by Tarnowski JR et al.[9], a 91-year old male patient with a proximal femoral fracture adjacent to the site of a stable hip arthroplasty was treated with reverse LISS and got good results.

Its biomechanics are inherently different from conventional plating techniques because of the fact that the latter require compression of the plate to the bone and rely on friction at the bone-plate interface. With increasing axial loading cycles, the screws can begin to toggle, which decreases the friction force and leads to plate loosening. If this occurs prematurely, fracture instability will occur, leading to implant failure (especially in metaphyseal and osteoporotic bone). This biomechanical prerequisite of conventional plates is associated with biological pitfalls due to compression of periosteal blood supply and compromise of the vascularity of the fracture. In contrast, LISS could avoid these problems.

Considering the biomechanical and biological advantages of the LISS, we try to treat intertrochanteric hip fractures which often occur in the elderly patients with osteoporosis by the use of the LISS plate in a reverse position. It has worked successfully as the definitive fixation device for this type of fracture.

The ease of use of the guide arm for screw placement and the ease of imaging the plate and screws are important advantages in the system. An important consideration in this technique is that a contralateral implant was used, i.e. a left sided implant was used for this right sided fracture. This allows for the anterior bowing of the femur and must be considered when using this technique. This does mean that there is occasionally a misfit between the implant and the greater trochanter, but this did not affect the fixation through some movement of implant as detailed.

The short operating time of this method is extremely important in the elderly patient where the other diseases may take precedence and rapid fracture fixation is required. The necessity to limit surgical time and modify operative intervention for the elderly patient is now well recognized.

The beneficial functional effect of less invasive surgery is also seen in patients with hip fractures. The mortality rate of zero percent during a hospital stay in the current study is compared with an early (within four weeks) mortality rate of 6.7 percent in a Gamma nail group and 9.4 percent in a dynamic hip screw group[10].

We are also aware of some limitations of our study. First, because of the nature of this retrospective study, we had no control group treated with alternative fixation procedures. Second, no functional outcomes were presented. Longer-term outcome analysis will be necessary. Third, the current study has only 28 patients; therefore, significant statistical conclusions are more difficult to make.

## Conclusions

Reverse LISS plating was used in our institution to treat stable and unstable intertrochanteric fractures. The results, particularly with the unstable intertrochanteric fractures, are encouraging because no fracture collapses were observed. As such, the Reverse LISS plating could present an additional treatment alternative for this fracture type.

## Competing interests

The authors declare that they have no competing interests.

## Authors' contributions

CQ Z led the design of the study and performed the operation, Y S wrote the paper, C Y, SB Cparticipated in data management and led the statistical analysis. DX J carried out the operation. BF Zcritically commented upon the paper. All authors read and approved the final manuscript.

## Pre-publication history

The pre-publication history for this paper can be accessed here:

http://www.biomedcentral.com/1471-2474/11/166/prepub
